# The canine isolate *Lactobacillus acidophilus* LAB20 adheres to intestinal epithelium and attenuates LPS-induced IL-8 secretion of enterocytes *in vitro*

**DOI:** 10.1186/s12866-014-0337-9

**Published:** 2015-01-16

**Authors:** Veera Kainulainen, Yurui Tang, Thomas Spillmann, Susanne Kilpinen, Justus Reunanen, Per EJ Saris, Reetta Satokari

**Affiliations:** Department of Veterinary Biosciences, Faculty of Veterinary Medicine, University of Helsinki, P.O. Box 66 (Agnes Sjöberginkatu 2), FI-00014 Helsinki, Finland; Department of Food and Environmental Sciences, Faculty of Agriculture and Forestry, University of Helsinki, P.O. Box 56 (Viikinkaari 9), FI-00014 Helsinki, Finland; Department of Equine and Small Animal Medicine, Faculty of Veterinary Medicine, University of Helsinki, P.O. Box 57 (Viikintie 49), FI-00014 Helsinki, Finland

**Keywords:** *Lactobacillus*, Probiotics, Adhesion, Anti-inflammatory, Transepithelial resistance, Interleukin-8, Canine

## Abstract

**Background:**

For a good probiotic candidate, the abilities to adhere to intestinal epithelium and to fortify barrier function are considered to be crucial for colonization and functionality of the strain. The strain *Lactobacillus acidophilus* LAB20 was isolated from the jejunum of a healthy dog, where it was found to be the most pre-dominant lactobacilli. In this study, the adhesion ability of LAB20 to intestinal epithelial cell (IECs) lines, IECs isolated from canine intestinal biopsies, and to canine, porcine and human intestinal mucus was investigated. Further, we studied the ability of LAB20 to fortify the epithelial cell monolayer and to reduce LPS-induced interleukin (IL-8) release from enterocytes.

**Results:**

We found that LAB20 presented higher adhesion to canine colonic mucus as compared to mucus isolated from porcine colon. LAB20 showed adhesion to HT-29 and Caco-2 cell lines, and importantly also to canine IECs isolated from canine intestinal biopsies. In addition, LAB20 increased the transepithelial electrical resistance (TER) of enterocyte monolayers and thus strengthened the intestinal barrier function. The strain showed also anti-inflammatory capacity in being able to attenuate the LPS-induced IL-8 production of HT-29 cells.

**Conclusion:**

In conclusion, canine indigenous strain LAB20 is a potential probiotic candidate for dogs adhering to the host epithelium and showing intestinal barrier fortifying and anti-inflammatory effects.

**Electronic supplementary material:**

The online version of this article (doi:10.1186/s12866-014-0337-9) contains supplementary material, which is available to authorized users.

## Background

The mammalian gastrointestinal tract (GIT) is colonized by a complex microbiota, which interacts with the host mucosa and maintains mucosal homeostasis in healthy individuals [1,2]. Studies on mice model have shown that the initial development of immune system is profoundly influenced by the colonization of gut microbiota [3]. The deficiency of immune maturation and the development of intolerance towards commensal bacteria may lead to chronic inflammatory diseases later in life [1,3]. The maintenance of intestinal immune and physiological homeostasis in mammals is mediated by sophisticated interaction between intestinal microbiota and the host mucosa, which consists of the epithelial cell layer and underlying lamina propria. The epithelial cell layer is not merely a physical barrier, but also responds to stimuli, e.g. by secreting mucus and inducing innate and adaptive immune responses [4,5]. Intestinal epithelial cells (IECs) express several pattern recognition receptors (PRRs) and can detect microorganism-associated molecular patterns (MAMPs) and subsequently release effector cytokines. Thus, IECs are important players in orchestrating tolerance or inflammatory responses against the resident microbiota. Commensal microbes have often developed a mutualistic relationship with the host and induce tolerogenic or immunoregulatory responses in the host [4].

Adhesion is considered as a crucial step for intestinal bacteria to colonize and further interact with the host epithelium and immune system. Intestinal bacteria can adhere to mucus, or bind to exposed IECs via their surface structures [6-9]. *Lactobacillus* species can be found along the mammalian GIT with various counts, typically being more dominant in the proximal small intestine [10]. The majority of strains utilized as probiotics belong to the genus *Lactobacillus* and a number of strains have been shown to adhere strongly to the epithelium [11-15]. Probiotics have been shown to modulate immune responses, being able to competitively exclude pathogenic bacteria, and enhance epithelial barrier functions [4]. Promising results have been obtained also in alleviating gastrointestinal disorders [16].

In response to enteropathogen infection, the intestinal epithelium releases proinflammatory molecules to recruit immune cells and induce an acute inflammatory response. Inflammation is an essential physiological response to infection and tissue protection, but non-regulated inflammatory responses can give rise to tissue injury and chronic disease [1,16]. Interleukin-8 (IL-8) is one of the key chemokines, which is responsible for the initiation of inflammatory cascades and recruitment of neutrophils into the mucosa [17]. It is considered, that after the acute inflammation, commensal bacteria have a key role in providing regulatory immune stimuli to extinguish the inflammation back to basal level [1]. Also probiotics have been demonstrated to suppress mucosal inflammation and restore cytokine balance towards an anti-inflammatory state [18-22].

*Lactobacillus acidophilus* strain LAB20 was isolated from canine small intestine, where it was found to be among predominant jejunal lactobacilli [23]. Further, administered LAB20 could persist in the dog gut for more than six weeks post-administration [24], which is a remarkably long period for a probiotic strain. Typically, probiotics are cleared from the majority of patients within weeks after the administration is terminated [25,26]. In the present study, we investigated the interaction of LAB20 with the host epithelium. We studied it’s adhesion ability to canine mucus and IECs from different compartment of dog intestine and to IEC lines. Further, we assessed the ability of LAB20 to attenuate LPS-induced IL-8 release from IECs and to fortify epithelial barrier function.

## Results

### Adhesion to mucus

**Figure 1:**
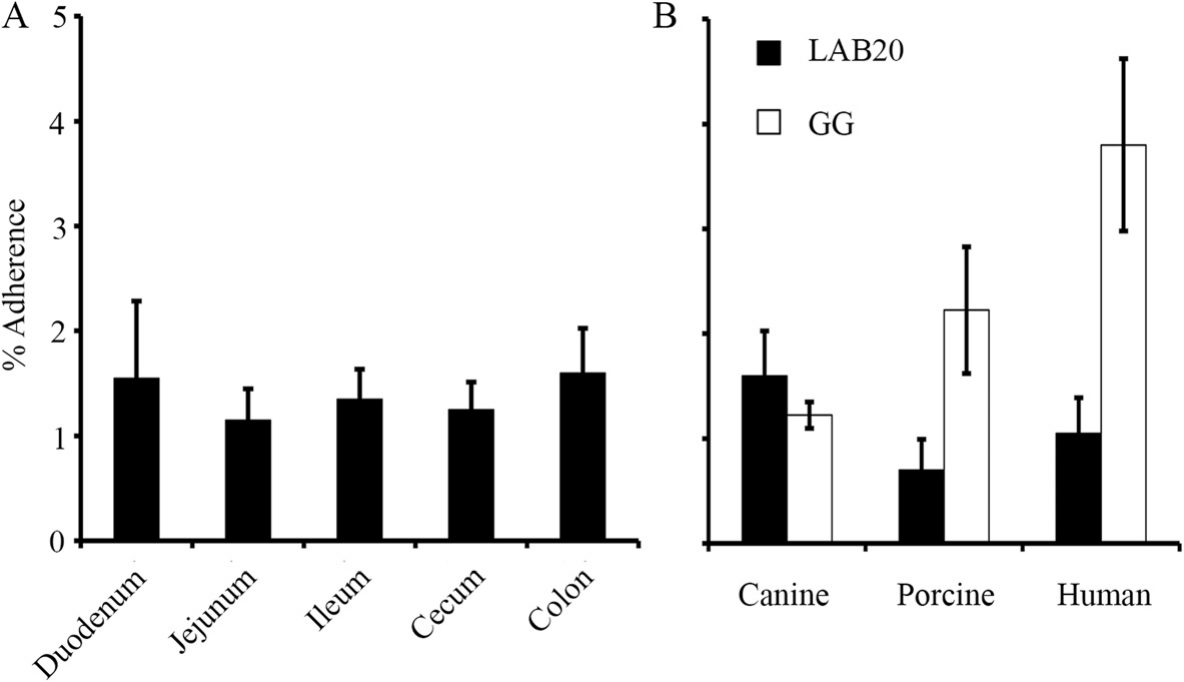
**Adhesion of** ***L. acidophilus*** **LAB20 to mucus.** Adhesion (%) of ^3^H-labeled LAB20 to mucus isolated from canine duodenum, jejunum, ileum, cecum, and colon **(A)** and to colonic mucus from different hosts **(B)** was measured. The human derived strain ^3^H-labeled *L. rhamnosus* GG was included for comparison. Results are the means ± standard deviations of five technical replicates (parallel wells) of the representative experiments.

First, we studied the ability of *L. acidophilus* LAB20 to bind mucus isolated from duodenum, jejunum, ileum, cecum and colon of canine intestine. We found that LAB20 showed very similar adhesion efficiency to canine mucus despite the mucus type i.e. from which intestinal compartments the mucus was isolated from (Figure 1A). Next, the capacity of LAB20 to adhere canine, human and porcine colonic mucus was compared. Human derived strain *Lactobacillus rhamnosus* GG (LGG), which has previously been shown to bind to human colonic mucus [7,27] was included in the experiment for comparison. LAB20 presented statistically significantly higher adhesion to canine colonic mucus (1.6%) compared to adhesion to porcine (0.7%) mucus (p < 0.05, Figure 1B). However, the binding of LAB20 to human (1.0%) mucus was not significantly different from adhesion to canine mucus. LGG adhered to human (3.8%) and porcine (2.2%) mucus more efficiently than LAB20 (p < 0.05), whereas LAB20 showed higher adhesion to canine mucus (Figure 1B).

### Adhesion to IECs

**Figure 2:**
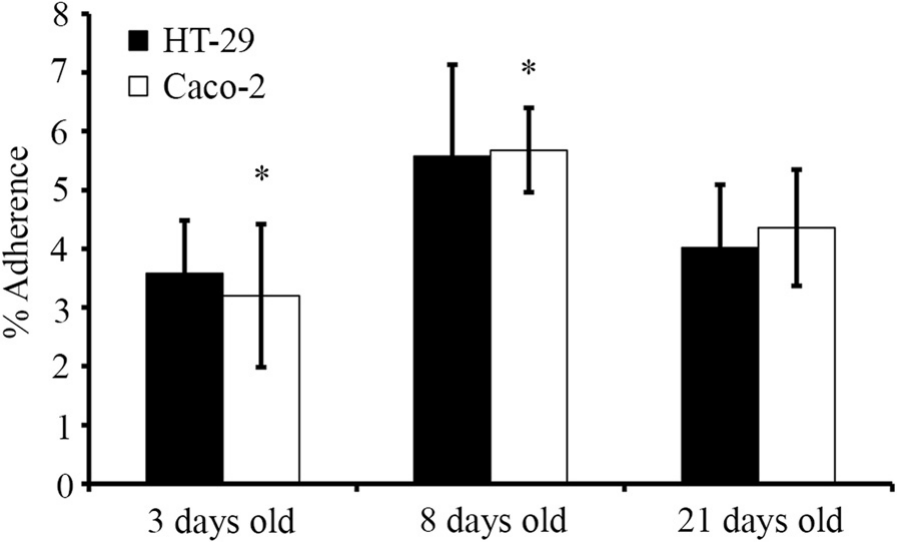
**Adhesion of LAB20 to epithelial cell lines.** Adhesion (%) of ^3^H-labeled *L. acidophilus* LAB30 to 3-, 8-, and 21-day-old Caco-2 and HT-29 cells was measured. The results of five technical replicates (parallel wells) from the representative experiment are expressed as means ± standard deviations. Significant reduction (p < 0.05) in adhesion compared to different aged IECs is indicated with an asterix.

**Figure 3:**
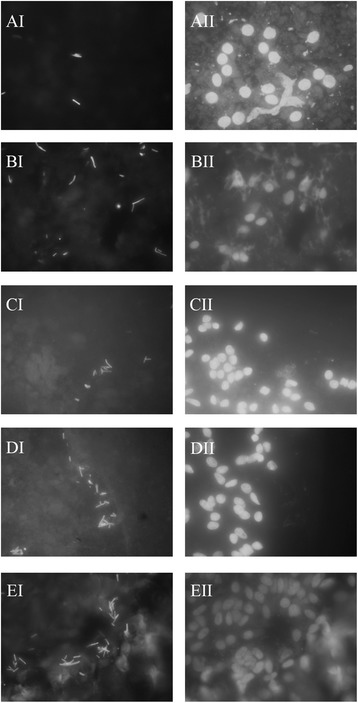
**Adhesion of LAB20 to the canine IECs.** Adherence of FITC-labelled bacteria to canine IECs obtained from duodenum **(A)**, jejunum **(B)**, ileum **(C)**, cecum **(D)**, and colon **(E)** sections is shown in the left panel. The arrows indicate LAB20 cells adhered to IECs. The nucleus of IECs were stained with DAPI and are shown in the right column.

Next, we studied the adherence of LAB20 to the epithelial cell lines of different ages. Caco-2 cells differentiate in 14 days after confluence [28], and the adhesion was examined with undifferentiated cells (3 days) in addition to cells at two differentiation stages (8 and 21 days). For comparison, the same growth times were used for HT-29 cell line. LAB20 adhered similarly to Caco-2 and HT-29 cell lines and there was no statistically significant difference in the adherence of LAB20 to IECs of different ages except the adhesion to 3-days old Caco-2 cells was significantly lower compared to 8-days old but not to 21-days old Caco-2 cells (Figure 2). In order to examine the adhesion of LAB20 to canine IECs, FITC-labeled LAB20 cells were visualized with epifluorescence microscopy. LAB20 adhered to canine IECs isolated from various parts of the intestine (Figure 3). The adherence to IECS isolated from cecum and colon seemed higher than to IECs from duodenum, jejunum or ileum, but the assay did not allow proper quantitative measurement and therefore, this difference remains unconfirmed.

### Ability to attenuate LPS-induced IL-8 production of enterocytes

**Figure 4:**
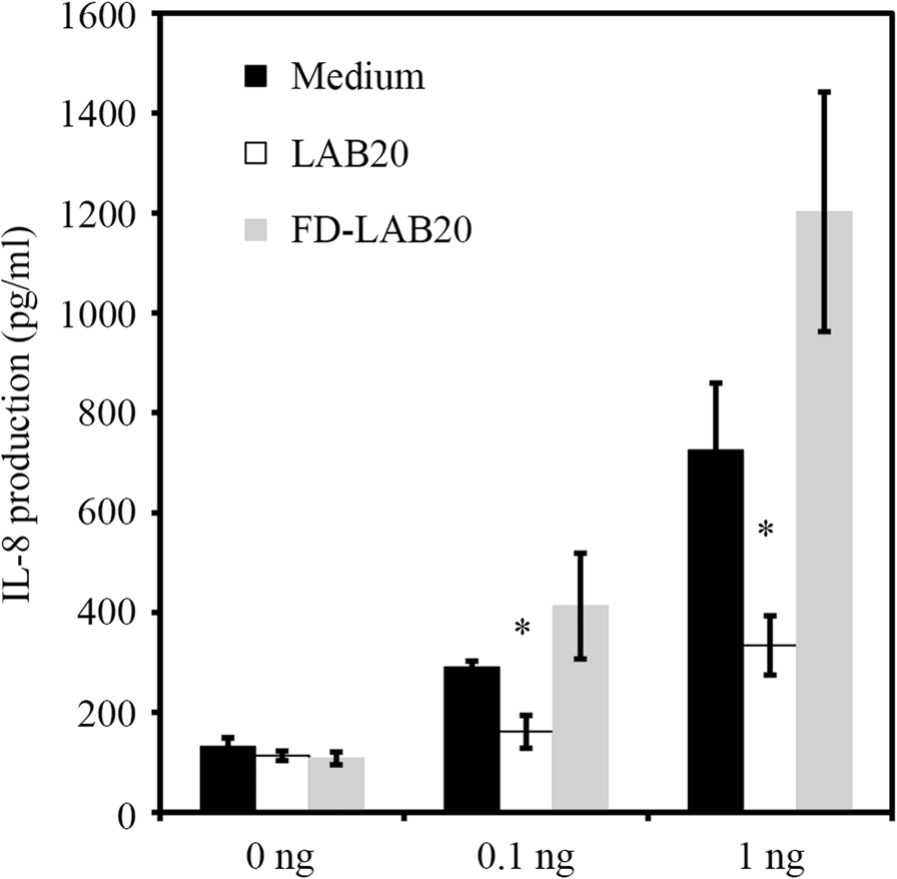
**The LPS-induced IL-8 production in HT-29 cells in response to medium, LAB20 cells, and freeze-dried LAB20 (FD-LAB20).** Data are expressed as means ± standard deviations of three technical replicates from a representative experiment. Significant difference (p < 0.05) in the IL-8 production as compared to the medium control is indicated with an asterisk.

In order to assess the potential anti-inflammatory properties of LAB20, we measured its ability to reduce the LPS-induced release of IL-8 from the HT-29 cell line. The attenuation effect on IL-8 production was evaluated by incubating HT-29 monolayer with LPS (0.1 or 1 ng/ml) after the cell line was first exposed to fresh and freeze-dried LAB20 and sterile culture medium (Figure 4). Prior co-incubation of HT-29 cells with fresh LAB20 decreased significantly (P < 0.05) the LPS-stimulated IL-8 production with both LPS concentrations. However, the freeze-dried LAB20 did not show reduction of secreted IL-8 as compared to the freshly cultured LAB20 cells. Taken together, the results showed that the decrease of IL-8 production, obtained by incubation with LAB20, was depended on the properties of active, living cells. LAB20 recovery from the freeze-dried stage takes more than 8 hours and thus we presumed it to remain inactive during the one hour incubation of attenuation experiment.

In order to disclose the molecular mechanisms behind the anti-inflammatory action of LAB20 we constructed a recombinant derivate strain, SAA658, to down-regulate the transcription of the exopolysaccharide synthesis *epsE* gene in LAB20 using antisense RNA strategy (Additional file 1). This strategy was chosen because knock-out mutants were not possible to construct due to the low transformation frequency (approximately 10 transformants μg^−1^ DNA) of LAB20. Our preliminary results suggest that exopolysaccharide (EPS) of LAB20 may have a role in the immunomodulatory activity of LAB20 (Additional file 1). However, the detailed properties of the derivate strain and the effector molecules of anti-inflammatory action still need to be resolved.

### Effects of LAB20 on epithelial barrier function

**Figure 5:**
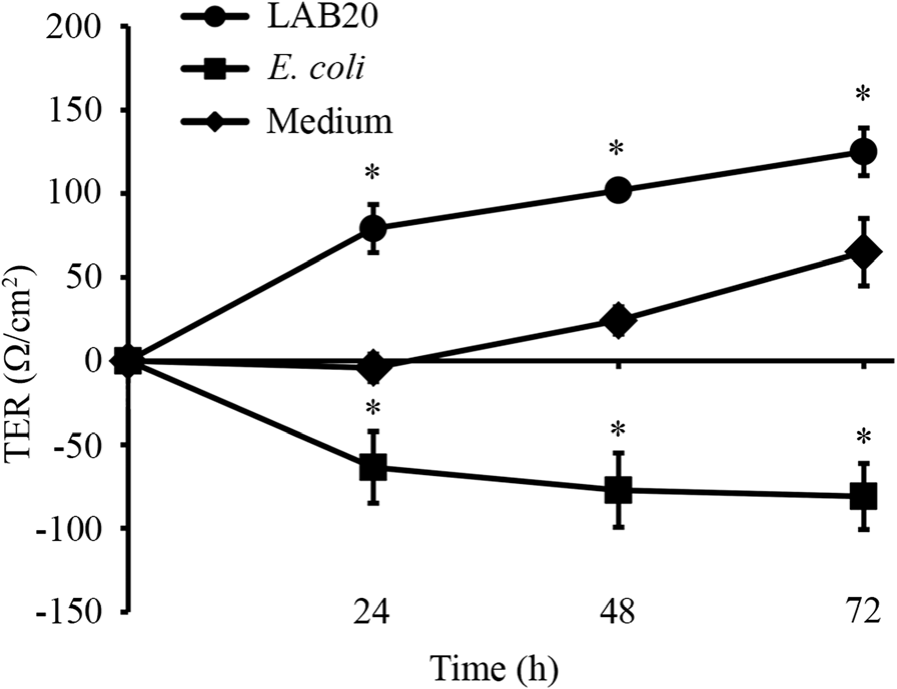
**The effect of LAB20 on transepithelial resistance (TER) of the Caco-2 cell line.** TER values (Ω/cm2) are the means ± standard deviations of three technical replicates from a representative experiment. Significant differences (p < 0.05) in TER-values between bacterium-treated and untreated Caco-2 cells are indicated with an asterix.

The effect of LAB20 on epithelial barrier function was studied by measuring transepithelial electric resistance (TER). Nonpathogenic *Escherichia coli* has been shown to disrupt the Caco-2 monolayer [29] and we included *E. coli* TOP10 in the experiment as a negative control. The epithelial cells were incubated with LAB20, *E. coli* or sterile culture medium for 72 hours and TER was measured every 24 hours. TER of epithelial cells treated with LAB20 was significantly higher as compared to cells treated with culture medium (Figure 5). In contrast, *E. coli* decreased TER significantly already within 24 hours.

## Discussion

Host-microbial interactions in the GIT are important for gut health, first by inducing the maturation of gut immune system in early life, and later in maintaining immune and physiological homeostasis of the mucosa [2,30-32]. It is considered that for an effective interaction with the host mucosa, bacteria must adhere to intestinal mucus or IECs. Adherence of probiotic bacteria to the intestinal epithelium is an important characteristic as it also promotes persistence time and colonization.

*L. acidophilus* LAB20 showed approximately 1.2-1.6% adhesion to canine mucus isolated from different intestinal compartments (Figure 1A) whereas the adhesion was lower to human- or porcine-derived mucus (Figure 1B). Instead, human-derived strain *L. rhamnosus* GG showed overall the highest adhesion ability to human colonic mucus. The results reinforce the suggestion that the adhesion of bacteria toward mucus is strain-specific, which may be due to the various bacterial surface structures associate with adhesion [7,11,27,33,34]. On the other hand, strains may display some degree of host specificity in the adhesion, although the strain origin is not always reflected in host specificity in adhesion [33,35].

The result showed that LAB20 could adhere to both Caco-2 and HT-29 cell lines, which are commonly used *in vitro* models to study microbe-IEC interactions [36]. Further, with access to canine IECs from different intestinal compartments, we presented that LAB20 could adhere to canine IECs as well. Canine intestinal epithelial cell lines are not commercially available and therefore, we used IECs isolated from duodenum, jejunum, ileum, cecum, and colon biopsies of healthy dogs. The gastrointestinal epithelium is covered with mucus, which forms a thick, continuous layer in the large intestine. However, in the small intestine, the mucus layer is thinner and discontinuous, allowing direct contact between epithelial cells and luminal bacteria. Also, under certain conditions, the mucus barrier is reduced, and bacteria can penetrate the layer and adhere to the underlying epithelial cells [37]. The canine jejunal isolate, LAB20, was previously found to persist in the gut of healthy dog for over six weeks after cessation of administration [24]. The long persistence in the canine GIT might be due to the effective adhesion to host IECs in the small intestine.

LAB20 was originally isolated from healthy canine jejunum, which makes it intriguing to it’s potential immunomodulatory effects. Canine primary colonic epithelial cells isolated from normal dogs express TLR4 that can be stimulated in response to LPS [38]. On the other hand, increased expression of TLR4 has been linked to inflammatory bowel disease (IBD) in dogs [39-41]. Further, LPS-carrying Proteobacteria are increased in human IBD subjects [42], and this bacterial phylum and LPS may play a role also in canine IBD. In this study, we used LPS-induced inflammation in HT-29 cell line as a model, because HT-29 cells express TLR4 that mediates LPS stimulation in IECs [43]. We found that LAB20 is able to attenuate LPS-induced release of IL-8 from HT-29 cell line. Previously, it has been shown that the stimulation of IECs with one MAMP can induce tolerance towards other MAMPs and modulate the inflammatory response of IECs [44]. This is a likely scenario also in the case of the attenuation of LPS-induced IL-8 secretion by LAB20. The result indicates that LAB20 could balance the IL-8 expression of enterocytes in response to apical stimulation by LPS from Gram-negative bacteria in the intestine. Most of the LPS-carrying bacteria in the GIT are harmless commensals. However, if the host responses to them are inappropriate or exaggerated, chronic inflammation (without infection) may ensue. There is growing evidence that aberrant innate immune responses towards the gut microbiota play a role in the pathogenesis of canine IBD [39,40]. Importantly, TLR4 expression is increased in dogs suffering from chronic enteropathies including IBD [39-41]. Thus, LAB20 seems to be able to induce in IECs tolerogenic response towards LPS derived from the intestinal microbiota.

Previously, bifidobacteria and *Lactobacillus fermentum* have been demonstrated to inhibit LPS or *Yersinia enterocolitica* –induced IL-8 production by IECs [45,46]. Moreover, several studies have shown that bifidobacteria and lactobacilli can reduce the severity of inflammation *in vivo* in rodent models and patients with IBD [18,19,47]. Our preliminary results suggest that EPS of LAB20 may have a role in the immunomodulatory activity of LAB20 (Additional file 1). *Lactobacillus amylovorus* inhibits the TLR4 inflammatory signaling triggered by enterotoxigenic *E. coli* and TLR2 is required for the suppression of TLR4 signaling activation [48]. Further, EPS of *Lactobacillus delbrueckii* have been shown to attenuate enterotoxigenic *E. coli* -induced inflammatory response in porcine IECs and TLR2 plays a central role in the immunomodulatory action [49]. Concerning LAB20, further studies are needed to confirm the anti-inflammatory properties *in vivo* in dogs.

One of the proposed mechanisms of action of probiotic LAB is the ability to strengthen the epithelial barrier [50]. We measured Caco-2 cell monolayer’s resistance, TER, as an indicator of GI-epithelial barrier function. Co-culture of Caco-2 cells with LAB20 fortified epithelial barrier, as demonstrated by the increase in TER. The Caco-2 cell line is a well-characterized model of the gut epithelium and is capable of differentiation and polarization [51] and measuring TER of polarized cell monolayers is commonly used as a screening assay to test for probiotic effects [45,52-54]. As expected, the non-pathogenic *E. coli* used in the experiment as a control disrupted the barrier integrity [29,55]. Thus, LAB20 can fortify intestinal barrier function by tightening the epithelial cell layer and inducing tolerance towards LPS.

## Conclusions

In conclusion, we demonstrated that canine derived strain LAB20 has potential as a probiotic as it adheres to mucus and IECs and interact with the host. Specifically, it fortifies epithelial cell layer, and is able to elicit anti-inflammatory responses in enterocytes. The anti-inflammatory property appeared to be associated with cell viability and activity, but the precise mechanisms, especially the components responsible for anti-inflammatory functions remain to be identified.

## Methods

### Microorganisms and growth condition

*L. acidophilus* LAB20 was previously isolated from canine jejunal chime [23]. It was cultured in LBS broth (BBL, Becton Dickinson) without acetic acid and pH adjusted to 7 with 5M NaOH (mLBS7) to optimize LAB20 growth, and incubated at 37°C in anaerobic conditions. Freeze-dried LAB20 cells were prepared as described previously [24]. The viability of freeze-dried LAB20 is 8% after freeze-drying protocol and the recovery of freeze-dried LAB20 takes more than 8 hours. *L. rhamnosus* GG was cultured in MRS (Becton Dickinson) broth under static conditions at 37°C and *E. coli* TOP10 was cultured in Luria-Bertani broth (Becton Dickinson) at 37°C.

### HT-29 cell and Caco-2 cell cultures

The human intestinal cell lines HT-29 and Caco-2 were obtained from DSMZ and were grown at 37°C in a 95% air–5% CO_2_ atmosphere. HT-29 cells were grown in McCoy 5A medium (Lonza) supplemented with 10% heat-inactivated (56°C, 30 min) fetal calf serum (FCS, Integro B.V.) and 100 U/ml penicillin-streptomycin (Lonza). Caco-2 cells were grown in RPMI 1640 medium (Sigma-Aldrich) supplemented with 2 mM _L_-glutamine (Lonza), 20% FCS, 100 U/ml penicillin-streptomycin, 15 mM HEPES (Lonza), and 1% nonessential amino acids (Lonza).

### Isolation of human and canine intestinal mucus

Human colonic mucus was collected from a healthy piece of tissue obtained from patients with colorectal cancer, by following the previous description [56]. The use of resected human intestinal tissue for the adhesion studies was approved by the ethical committee of the Hospital District of Southwest Finland and the patients donating their samples for research purposes signed a written informed consent. Briefly, the mucus was collected into HEPES-Hanks buffer (10 mM HEPES, pH 7.4) by gently scraping with a rubber spatula from washed resected material (PBS containing 0.01% gelatin), and stored at −20°C until use. Canine intestinal mucus was isolated from intestinal mucosa samples taken by necropsy from the duodenum, jejunum, ileum, cecum, and colon of six healthy dogs being euthanized after finalizing an unrelated experimental study. The study protocol was approved by the Finnish National Animal Experiment Board (License number ESAVI-2010-04178 Ym-23, PH 1465A). Fresh mucosa was isolated from the intestinal wall with the back side of a scalpel after thorough cleaning of the surface with cold saline. All samples were immediately frozen in liquid nitrogen and stored at −80°C until being melted at room temperature for further processing. For the adhesion tests the samples were carefully centrifuged and the mucus and epithelial cells were separated.

### Bacterial adhesion to mucus and cell lines

For the adhesion test, mucus samples (50 μg in PBS) were immobilized passively on Maxisorp microtiter wells by overnight incubation at 4°C [56]. The wells containing immobilized mucus were washed twice with PBS and incubated with blocking buffer (0.5% [w/v] BSA in PBS) for one hour at room temperature. The Caco-2 and HT-29 cell lines were cultivated on 96-well tissue culture plate (10,000 cells/well; Nunc) for 3, 8 and 21 days. The cells were washed twice with culture medium before the adhesion assay. LAB20 was metabolically radiolabeled by cultivating bacteria with 10 μl/ml [5′-^3^H] thymidine (17.0 Ci/mmol; Perkin Elmer). The adhesion assay was performed as described previously by Kainulainen et al. (2013) [57]. Briefly, after cultivation, bacteria were collected by centrifugation and washed with McCoy 5A (adhesion to HT-29) or RPMI (adhesion to Caco-2) without supplements, or PBS (adhesion to mucus). The optical density was adjusted (OD600_nm_ = 0.25) to the same culture medium or buffer used for washing. Bacteria (100 μl) were incubated on mucus at 37°C or on the IECs in a CO_2_ incubator at 37°C for one hour, and the non-adherent bacteria were removed by washing the wells three times with PBS. Bacteria bound to cells or mucus were lysed with 1% SDS–0.1 M NaOH by incubating at 60°C for one hour. The radioactivity of the suspension was measured by liquid scintillation. Four to five parallel wells (i.e. technical replicates) were used in each experiment, and all experiments were repeated two to four times. The percent bacterial adhesion was determined by calculating the ratio between the radioactivity of the adherent bacteria and that of the added bacteria.

### Bacterial adhesion to canine IECs

Canine IECs were isolated from canine mucosa samples by separating the cells from mucus by centrifugation. The IECs from duodenum, jejunum, ileum, cecum, and colon were mounted on glass slides and fixed with 4% (w/v) paraformaldehyde (Sigma-Aldrich) in PBS (pH 7.4) for overnight at 4°C. For the adhesion assay, a 1.5-ml volume of LAB20 cells from two-night culture were collected by centrifugation and washed twice with PBS, then stained with 2.5 ml of 75 μg/ml of fluorescein isothiocyanate (FITC; Sigma-Aldrich) in PBS (pH 8.2). After one hour incubation at room temperature in dark bacterial cells were washed with 0.01% Tween20-PBS (pH 7.4), and the optical density was adjusted to OD600_nm_ = 0.25 with the same buffer used for washing. The FITC-labelled bacteria were incubated with canine IECs for one hour at room temperature in a moisture chamber to allow bacteria to bind. The slides were then washed in 50 ml PBS for three times, and epithelial cells were stained with 10 μM 4,6'-diamidino-2-phenylindole (DAPI; Molecular Probes), which binds to nucleic acids. The adherent bacteria were examined with an epifluorescence microscopy (Leica DM 4000B) and images were digitally recorded using CellP^^^ imaging software for life sciences microscopy (Soft Imaging System GmbH). The assay was carried out twice with duplicate samples, and the figures are representative microscopic images of bacterial adherence.

### Induction of IL-8 release from HT-29 by LPS and the attenuation assay

LAB20 cells were harvested from 36–40 hours culture by centrifugation and freeze-dried LAB20 cells were prepared as described previously [24]. Cells were washed once with McCoy 5A medium with FCS. The optical density of bacterial suspensions was adjusted to 0.25 at 600 nm (OD 600), respectively. A 100 μl volume of each bacterial cell suspension was added to the wells containing 8-days-old HT-29 cells and incubated at 37°C for one hour in a 95% air–5% CO_2_ atmosphere. The control wells contained only McCoy 5A medium with FCS. Afterwards, the medium and bacterial suspension were removed from wells, and 200 μl of *E. coli* LPS-containing (0.1 ng/ml or 1 ng/ml) medium were added. The HT-29 cells were incubated with LPS for four hours, and the IL-8 concentration of the medium was measured by ELISA (Human IL-8 BD OptEIA™ Kit, BD).

### Transepithelial electric resistance

The effect of LAB20 on epithelial cell integrity was studied as previously described by Myllyluoma et al. [58] with minor modifications. Briefly, LAB20 and *E. coli* cells were harvested after cultivation by centrifugation and washed once with RPMI containing the supplements. The optical density was adjusted to OD_600nm_ = 0.25 with the same RPMI medium. Caco-2 cells (50 000 cells/well) were cultured for eight days on cell culture inserts (Millipore) with a 3-μm pore size inserted in the wells of 24-well culture plate. The adjusted bacterial suspension (400 μl) was added on the apical side of the insert and the cultures were incubated at 37°C under a 5% CO_2_ atmosphere for 72 h. Transepithelial electrical resistance (TER) across the monolayers was measured by using Millicell ERS-2 TER meter (Millipore) at 24, 48 and 72 h of incubation. Measurements are expressed as Ω/cm^2^ after subtracting the baseline mean resistance of the same inserts i.e. the resistance at time point 0.

### Statistical analysis

A pairwise Student’s *t* test was used to determine the significant difference (*P* < 0.05). Results are shown as means ± standard deviations for technical replicates from representative experiments.

## Additional file

Additional file 1:
**Construction of derivate strain SAA658 and comparison of WT-LAB20 and SAA658 in the IL-8 production assay.**
