# Supplementary material for: The canine isolate Lactobacillus acidophilus LAB20 adheres to intestinal epithelium and attenuates LPS-induced IL-8 secretion of enterocytes in vitro
Source: BMC Microbiol. 2015 Jan 16;15(1):4. doi: 10.1186/s12866-014-0337-9 (PMC4320479; doi:10.1186/s12866-014-0337-9)
Supplement: Additional file 1: — Construction of derivate strain SAA658 and comparison of WT-LAB20 and SAA658 in the IL-8 production assay. [file 12866_2014_337_MOESM1_ESM.pdf]

CONSTRUCTION OF SAA658 DERIVATE STRAIN AND COMPARISON OF WT-LAB20 AND SAA658 IN THE IL-8 PRODUCTION ASSAY

MATERIALS AND METHODS

Construction of mutant SAA658. The LAB20 competent cells were prepared according to method from Wei et al [1] with minor modifications. Briefly, in order to weaken the cell wall of LAB20, penicillin was added to LAB20 culture with final concentration of 10 µg/ml, when OD_{660} reached to 0.1-0.2. The cells were harvested after 100 min incubation at 37°C, washed twice in ice-cold washing buffer (5 mM sodium phosphate, pH 7.4, 1 mM $MgCl_2$), then resuspended to 1% of the original culture volume in ice-cold electroporation buffer (0.9 M sucrose, 3 mM $MgCl_2$, pH 7.4). Aliquots of 50 µl of the cell suspension were stored at -80°C.

Genomic DNA of *L. acidophilus* LAB20 was extracted according to the method described by Manninen et al. [2], and diluted 1/20 (vol/vol) to use as template for PCR. The partial *epsE* gene, with ribosomal binding site (RBS), was amplified with primer pair EAF and EAR (Additional file table 1.) from LAB20 genomic DNA using Phusion high-fidelity DNA polymerase (Thermo Scientific). The LAB20 surface layer (S-layer) protein promoter region was amplified with primers EPF and EPR (Additional file table 1). By using overlap PCR, two regions were combined with primer EPF and EAR. This fragment was cloned into vector pLEB579 at cloning sites of *Bss*HII and *Eco*RV for expression of antisense-RNA, and transformed into LAB20 competent cells (2 kv, 400 Ω, 8 ms). Transformants were selected from mLBS7 plate with 5 µl/ml Ery, and the DNA fragment was amplified and sequenced from these transformants. The transformant having the expected structure was named as SAA658 and stored in -80°C.

Additional file table 1. Primer sets and PCR conditions

Methods	Name	Sequence (5'-3')	Cycle conditions	Product size (bp)	
			Annealing	Extension	
PCR	EPF	AAAGCGCGCTGCTTGTGGGGGT	50°C, 30 s	30 s	105
	EPR	ATCATTTTTCCTCTTACCCTGATTCATATTGTACTAAC			
PCR	EAF	AGTACAATATGAATCAGGGTAAGAGGAAAAATGAT	55°C, 30 s	30 s	129
	EAR	TTTGATATCTGATAAACATACCGCCCATGC			
Overlap PCR	EPF	AAAGCGCGCTGCTTGTGGGGGT	55°C, 30 s	30 s	191
	EAR	TTTGATATCTGATAAACATACCGCCCATGC			

RESULTS

Comparison of SAA658 and wild type LAB20 in the attenuation of LPS-induced IL-8 production. The derivative strain SAA658 was constructed to alter the exopolysaccharide (EPS) production of LAB20 cells. EPS are long-chain polysaccharides composed of branched, repeating units of sugars or sugar derivatives loosely attached to the cell surface or secreted into the environment [3]. In order to compare the anti-inflammatory properties of SAA658 and LAB20, we measured their abilities to reduce the LPS-induced release of IL-8 from HT-29 cell line as described in the article´s main text. The attenuation effect on IL-8 production was evaluated by incubating

HT-29 monolayer with LPS (0.1 or 1 ng/ml) after the cell line was first exposured to LAB20 or SAA658. Prior co-incubation of HT-29 cells with LAB20 decreased significantly the LPS-stimulated IL-8 production with the higher LPS concentration ($P < 0.05$), whereas no significant reduction was obtained with SAA658 (Additional file figure 1).

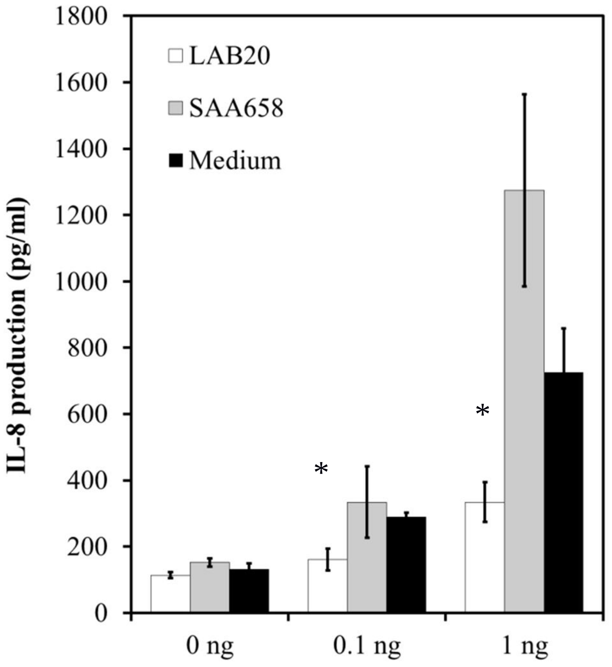

Additional file figure 1. The LPS-induced IL-8 production in HT-29 cells in response to medium, LAB20 cells and SAA658. Data are expressed as means ± standard deviations. Significant suppression ($P < 0.05$) of IL-8 production as compared to the medium control is indicated with an asterisk.

REFERENCES

1. Wei M, Rush CM, Norman JM, Hafner LM, Epping RJ, Timms P: **An improved method for the transformation of *Lactobacillus* strains using electroporation.** *J Microbiol Methods* 1995, **21** (1):97-109.

2. Manninen TJ, Rinkinen ML, Beasley SS, Saris PE: **Alteration of the canine small-intestinal lactic acid bacterium microbiota by feeding of potential probiotics**. *Appl Environ Microbiol* 2006, **72** (10):6539-6543.

3. Velez MP, de Keersmaecker SC, Vanderleyden J: **Adherence factors of *Lactobacillus* in the human gastrointestinal tract.** *FEMS Microbiol Lett* 2007, **276** (2):140-148.
